# Supplementary material for: Iron oxide nanozymes stabilize stannous fluoride for targeted biofilm killing and synergistic oral disease prevention
Source: Nat Commun. 2023 Sep 29;14:6087. doi: 10.1038/s41467-023-41687-8 (PMC10541875; doi:10.1038/s41467-023-41687-8)
Supplement: Supplementary file 1 — Supplementary Information File [file 41467_2023_41687_MOESM1_ESM.pdf]

Supplementary Information

Iron oxide nanozymes stabilize stannous fluoride for targeted biofilm killing and synergistic oral disease prevention

Yue Huang[1,2,3§], Yuan Liu[2,4§], Nil Kanatha Pandey[1,2,3§], Shrey Shah[1,5], Aurea Simon-Soro[2,3,6], Jessica C. Hsu[1,5], Zhi Ren[2,3,7], Zhenting Xiang[2,3], Dongyeop Kim[2,3,8], Tatsuro Ito[2,3,9], Min Jun Oh[2,3,10], Christine Buckley[11], Faizan Alawi[12], Yong Li[2,3], Paul J. M. Smeets[13,14], Sarah Boyer[14], Xingchen Zhao[14], Derk Joester[14], Domenick T. Zero[11], David P. Cormode[1,5]*, Hyun Koo[2,3,7]*

[1]Department of Radiology, Perelman School of Medicine, University of Pennsylvania, Philadelphia, PA, USA
[2]Biofilm Research Labs, Levy Center for Oral Health, School of Dental Medicine, University of Pennsylvania, Philadelphia, PA, USA
[3]Department of Orthodontics and Divisions of Pediatric Dentistry and Community Oral Health, School of Dental Medicine, University of Pennsylvania, Philadelphia, PA, USA
[4]Department of Preventive and Restorative Sciences, School of Dental Medicine, University of Pennsylvania, Philadelphia, PA, USA
[5]Department of Bioengineering, School of Engineering and Applied Sciences, University of Pennsylvania, Philadelphia, PA, USA
[6]Department of Stomatology, Dental School, University of Seville, Spain
[7]Center for Innovation and Precision Dentistry, School of Dental Medicine, School of Engineering and Applied Sciences, University of Pennsylvania, Philadelphia, PA, USA
[8]Department of Preventive Dentistry, School of Dentistry, Jeonbuk National University, Jeonju, Republic of Korea
[9]Department of Pediatric Dentistry, Nihon University School of Dentistry at Matsudo, Chiba, Japan
[10]Department of Chemical and Biomolecular Engineering, University of Pennsylvania, Philadelphia, PA, USA
[11]Department of Cariology, Operative Dentistry and Dental Public Health and Oral Health Research Institute, Indiana University School of Dentistry, Indianapolis, IN, USA
[12]Department of Basic and Translational Sciences, School of Dental Medicine, University of Pennsylvania, Philadelphia, PA, USA
[13]Northwestern University Atomic and Nanoscale Characterization Experimental Center, Northwestern University, Evanston, IL, USA
[14]Department of Materials Science and Engineering, Northwestern University, Evanston, IL, USA

*Corresponding Authors: Hyun Koo; Email: koohy@upenn.edu
David P. Cormode; Email: david.cormode@pennmedicine.upenn.edu

[§]These authors contributed equally to this work.

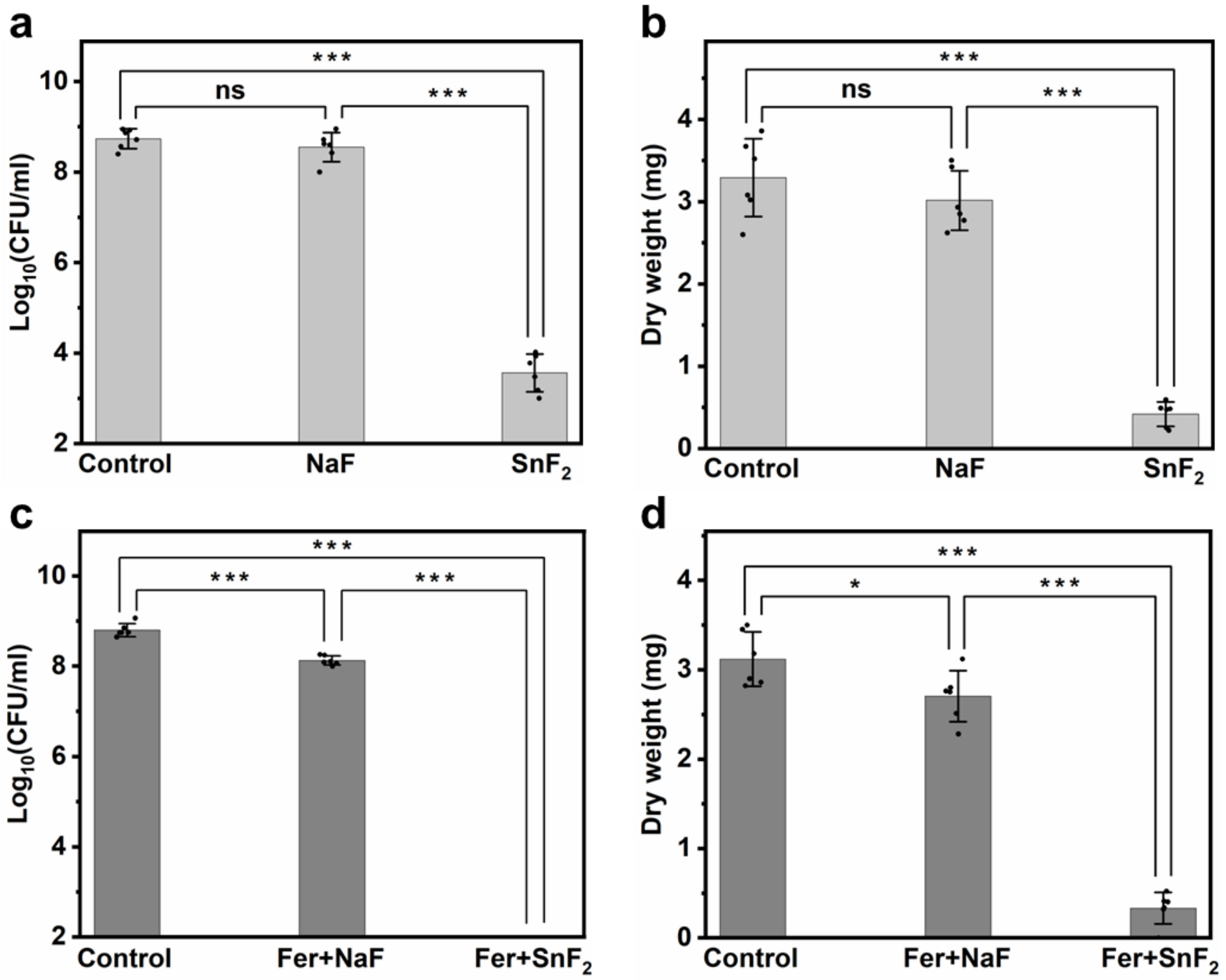

Supplementary Fig. 1. Antibiofilm studies of NaF and SnF_2 with or without Fer. The bacterial viability (**a**) and the mass of biofilm (**b**) after treatment with NaF or SnF_2 at 1000 ppm of F. The bacterial viability (**c**) and the mass of biofilm (**d**) after treatment with Fer+NaF or Fer+SnF_2 at 1 mg of Fe/ml, 1000 ppm of F, and 1% (v/v) of H_2O_2. The data are presented as mean ± standard deviation (n=6; 3 independent experiments with two replicates). *$p < 0.05$, ***$p < 0.001$; ns, nonsignificant; one-way ANOVA followed by Tukey test. Source data are available as a Source Data file.

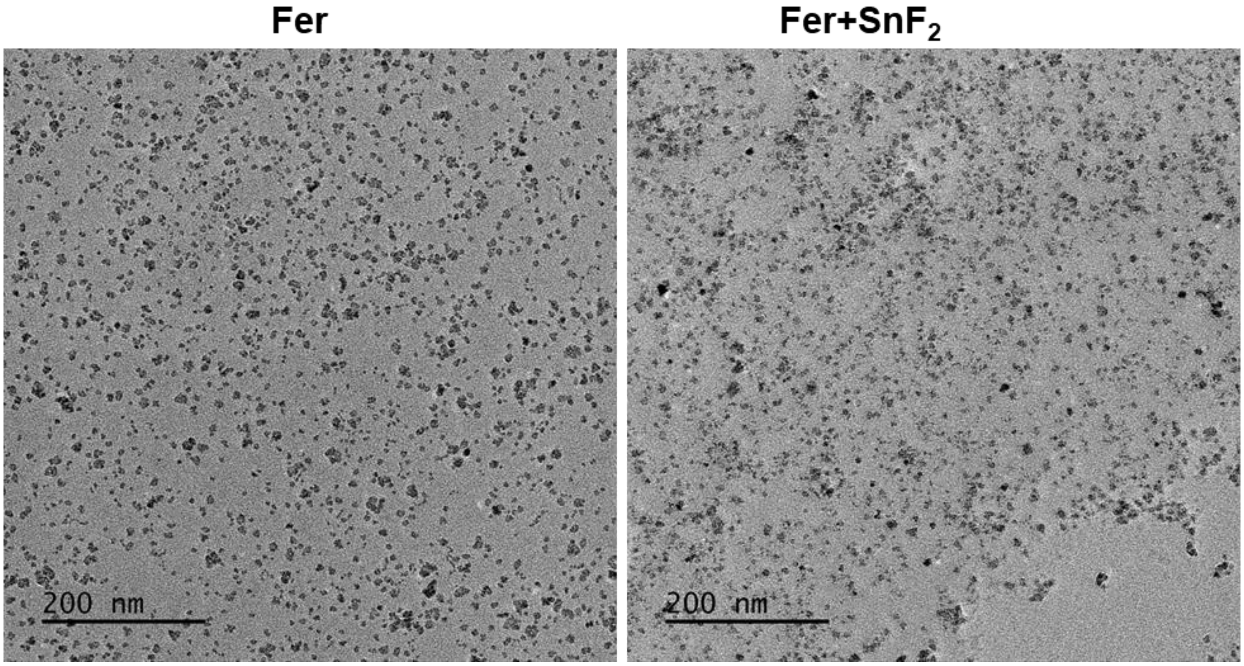

Supplementary Fig. 2. TEM of Fer and Fer+SnF_2. Representative TEM of Fer and Fer+SnF_2 after 1 h incubation in 0.1 M sodium acetate buffer (pH 4.5).

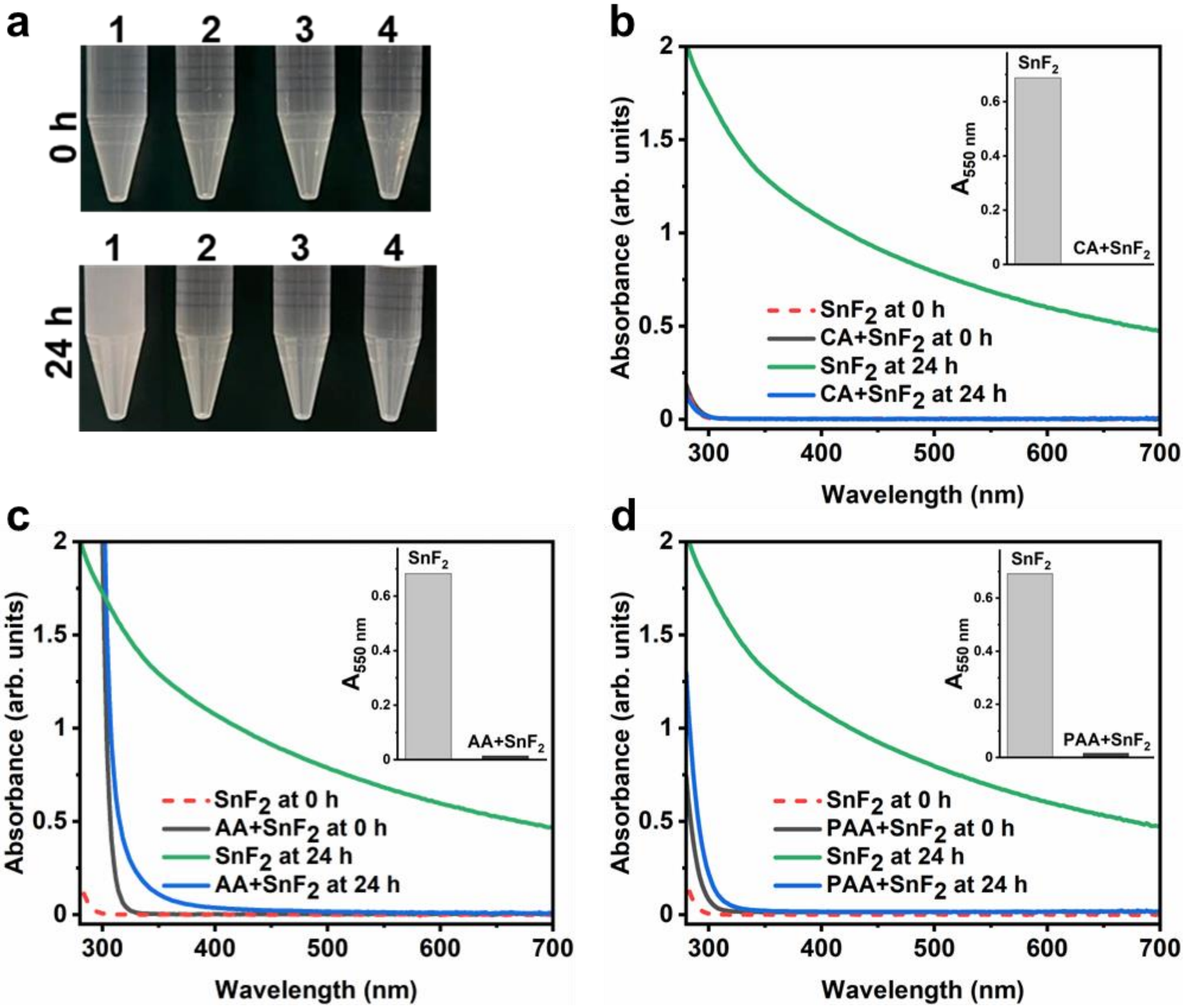

Supplementary Fig. 3. Stability study of SnF_2 when mixed with different chemicals. a Photographs of SnF_2 in different conditions at pH 4.5 (0.1 M sodium acetate buffer). The samples are: 1. SnF_2 alone, 2. citric acid (CA)+SnF_2, 3. L-ascorbic acid (AA)+SnF_2, and 4. poly(acrylic acid) (PAA)+SnF_2. Top: 0 h; bottom: after 24 h. **b-d** UV-visible absorption spectra of SnF_2 (250 ppm of F) at pH 4.5 (0.1 M sodium acetate buffer) with or without CA (1 mg/ml) (**b**), AA (1 mg/ml) (**c**), and PAA (1 mg/ml) (**d**) at the time points indicated. Insets show the absorbance of SnF_2 with or without CA (**b**), AA (**c**), and PAA (**d**) at 550 nm after 24 h incubation as a measure of turbidity. Source data are available as a Source Data file.

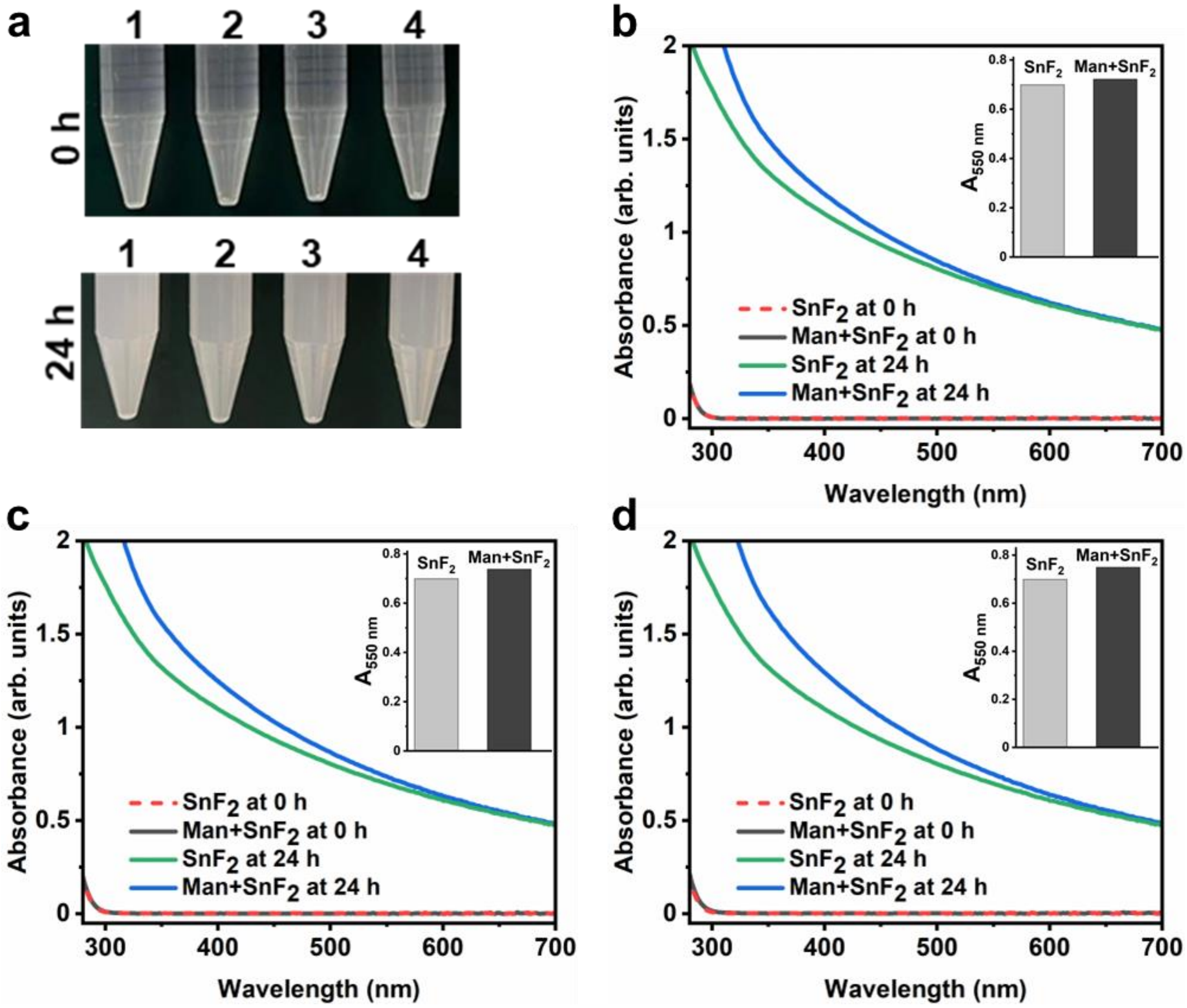

Supplementary Fig. 4. Stability study of SnF_2 in the presence of various amounts of mannitol. **a** Photographs of SnF_2 with various amounts of mannitol (Man) at pH 4.5 (0.1 M sodium acetate buffer) after 0 or 24 h incubation. The samples are: 1. SnF_2 alone, 2. 1 mg/ml of Man+SnF_2, 3. 2 mg/ml of Man+SnF_2, and 4. 10 mg/ml of Man+SnF_2. The concentration of SnF_2 was 250 ppm of F. Top: 0 h; bottom: after 24 h. **b-d** UV-visible absorption spectra of SnF_2 at pH 4.5 (0.1 M sodium acetate buffer) with or without 1 mg/ml (**b**), 2 mg/ml (**c**), and 10 mg/ml (**d**) of Man at the time points indicated. Insets show the absorbance of SnF_2 with or without 1 mg/ml (**b**), 2 mg/ml (**c**), and 10 mg/ml (**d**) of Man at 550 nm after 24 h incubation as a measure of turbidity. Source data are available as a Source Data file.

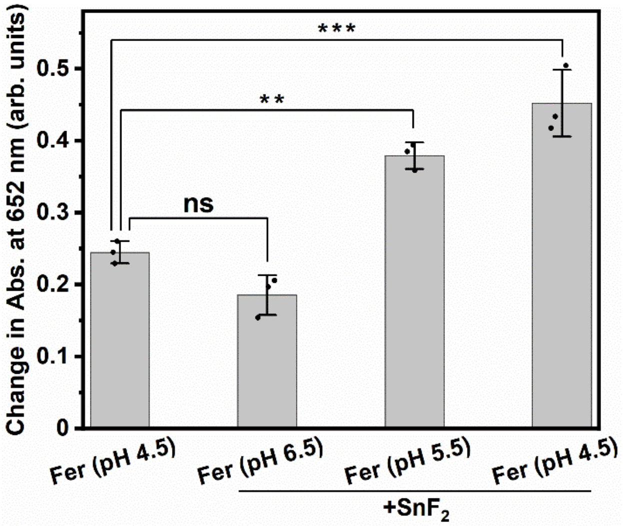

Supplementary Fig. 5. Comparison of catalytic activities of Fer+SnF_2 prepared at different pH values. Briefly, Fer was incubated with SnF_2 for 1 h at three different pH (4.5, 5.5, and 6.5; 0.1 M sodium acetate buffer) and washed three times with respective buffers using ultrafiltration tubes (3 kDa MWCO). Subsequently, the pellets were resuspended in a volume equal to the volume of the filtrate. All the catalytic activities were then determined (5 min incubation in the presence of 1% of H_2O_2) using TMB assay in 0.1 M sodium acetate buffer at pH 4.5. For the purpose of comparison, Fer alone (incubated at pH 4.5) was treated in a similar way. The data are presented as mean ± standard deviation (n=3 independent experiments). $**p < 0.01$, $***p < 0.001$; ns, nonsignificant; one-way ANOVA followed by Tukey test. Source data are available as a Source Data file.

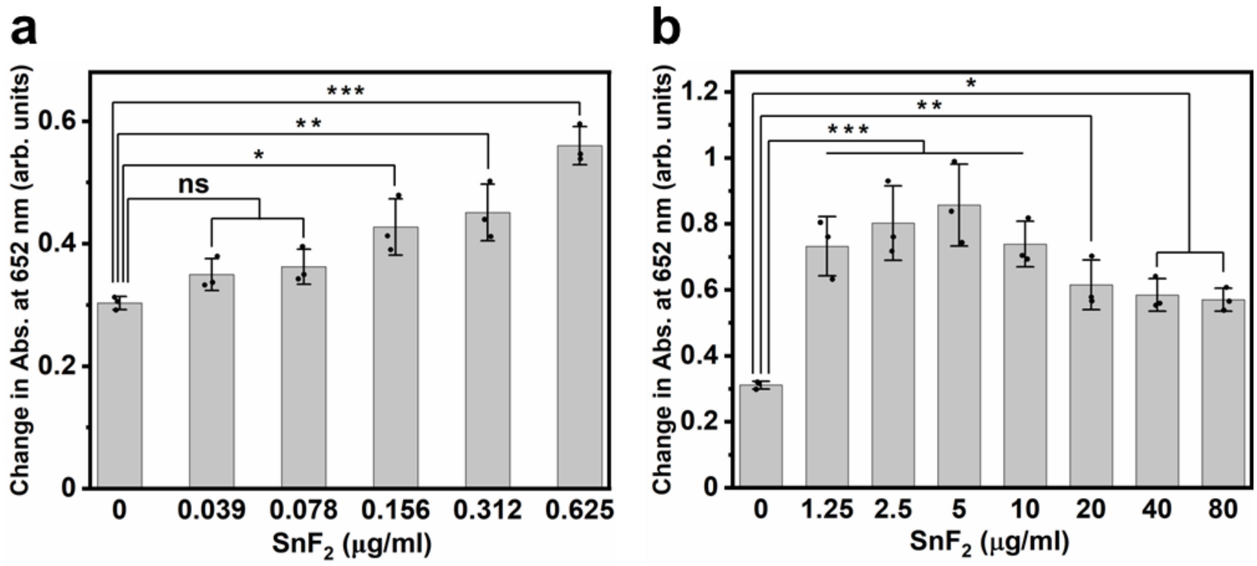

Supplementary Fig. 6. Study of various concentrations of SnF_2 on the catalytic activity of Fer. Effect of 0.039-0.625 µg/ml (**a**) and 1.25-80 µg/ml (**b**) of SnF_2 on the catalytic activity of Fer (20 µg of Fe/ml) in 0.1 M sodium acetate buffer (pH 4.5), as assessed by TMB colorimetric assay. 0 µg/ml indicates Fer alone. The data are presented as mean ± standard deviation (n=3 independent experiments). **p* < 0.05, ***p* < 0.01, ****p* < 0.001; ns, nonsignificant; one-way ANOVA followed by Tukey test. Source data are available as a Source Data file.

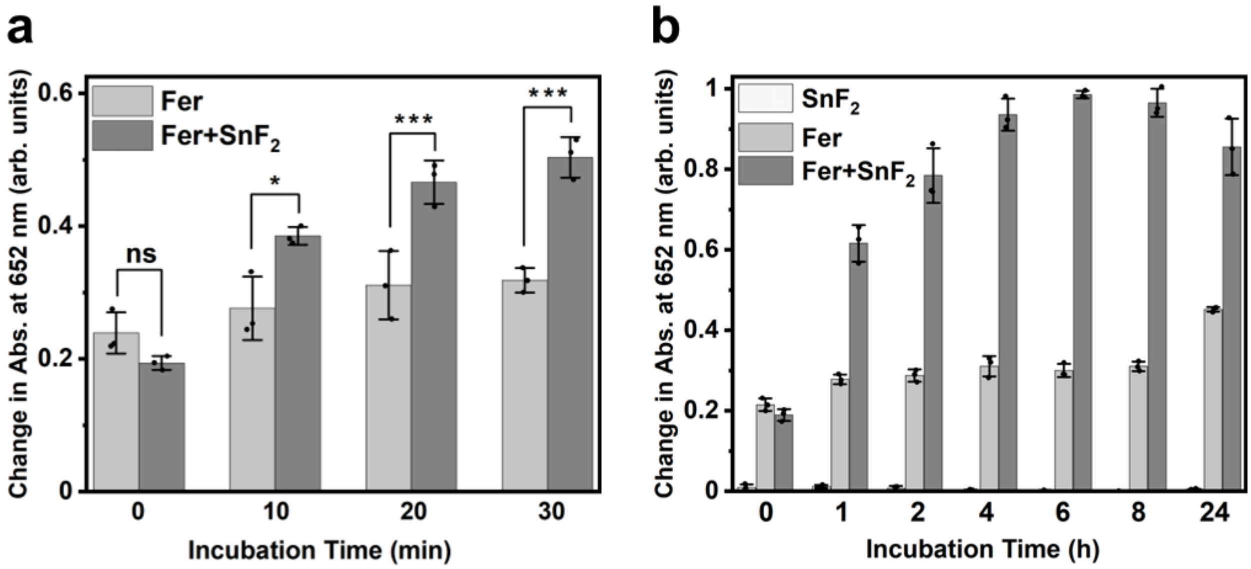

Supplementary Fig. 7. Evaluation of incubation time on the catalytic activity of Fer when mixed with SnF_2. a, b Effect of incubation time on the catalytic activity of Fer (20 µg of Fe/ml) with or without SnF_2 (20 µg/ml) in 0.1 M sodium acetate buffer (pH 4.5), as assessed by TMB colorimetric assay. The increase in absorption at 652 nm shows ROS production. The data are presented as mean ± standard deviation (n=3 independent experiments). *$p < 0.05$, ***$p < 0.001$; ns, nonsignificant; one-way ANOVA followed by Tukey test. Source data are available as a Source Data file.

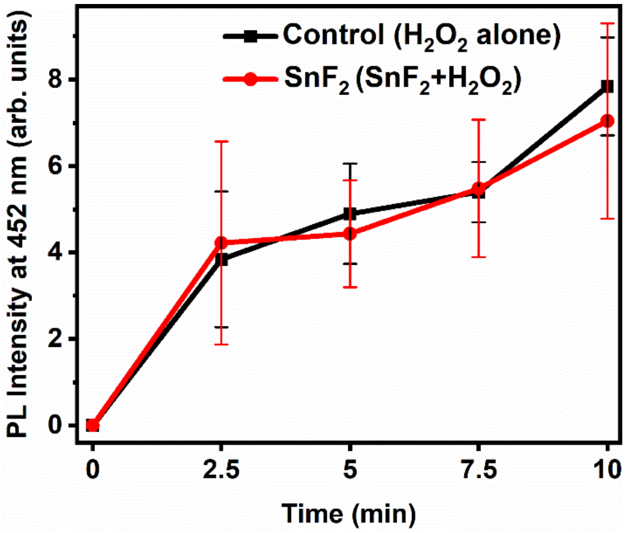

Supplementary Fig. 8. Evaluation of hydroxyl radical (•OH) production by SnF_2. Change in PL intensity of 7-hydroxycoumarin at 452 nm as a function of time with or without SnF_2 (20 µg/ml). The data are presented as mean ± standard deviation (n=5 independent experiments). Source data are available as a Source Data file.

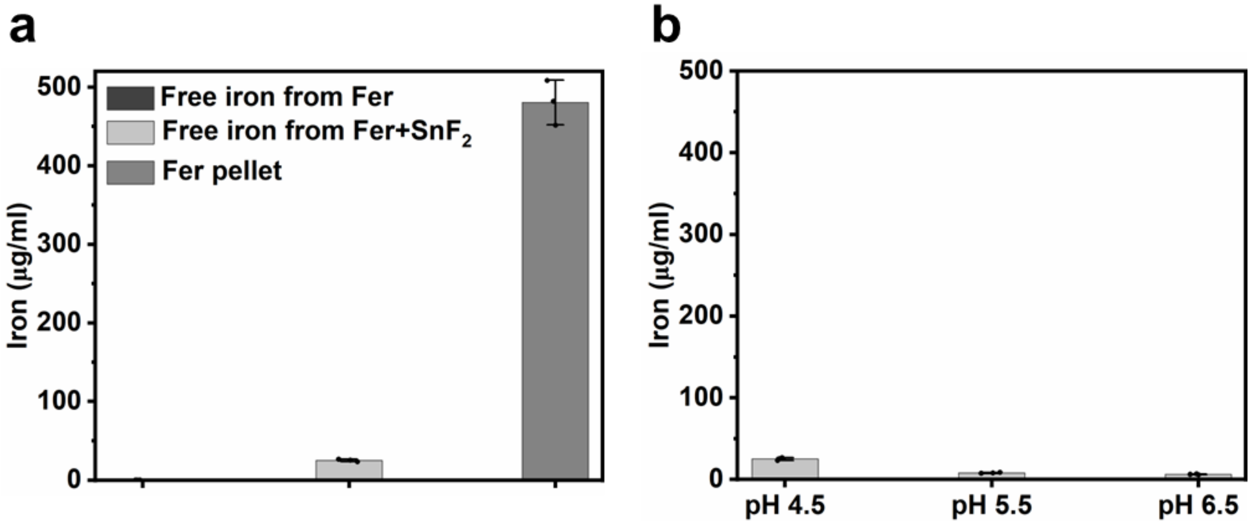

Supplementary Fig. 9. Iron release study. **a** Concentration of iron released from Fer (0.5 mg of Fe/ml) at pH 4.5 after 1 h incubation in the absence of SnF_2, presence of SnF_2 (0.5 mg/ml), and after the dissolution of the pellet in a volume equal to the volume of the filtrate, as determined by ICP-OES. **b** Concentration of iron released from Fer (0.5 mg of Fe/ml) in the presence of SnF_2 (0.5 mg/ml) during incubation at the indicated pH for 1 h. The data are presented as mean ± standard deviation (n=3 independent experiments). Source data are available as a Source Data file.

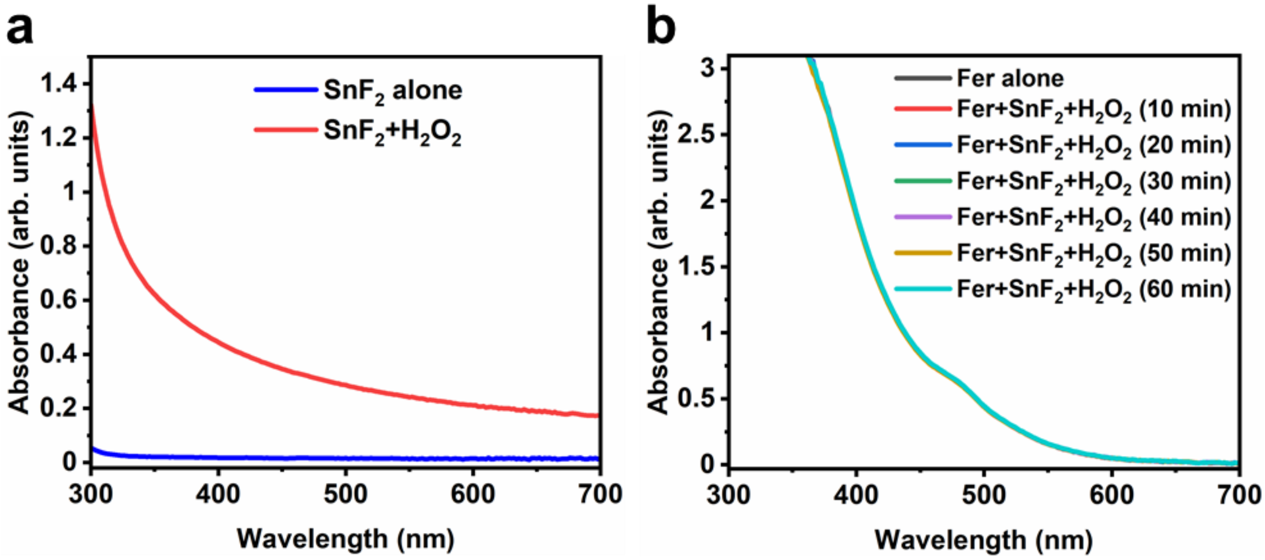

Supplementary Fig. 10. Stability study of SnF_2 with or without Fer after H_2O_2 treatment. a Representative UV-visible absorption spectra of SnF_2 with or without H_2O_2 (10 min incubation) in 0.1 M sodium acetate buffer (pH 4.5). The addition of H_2O_2 resulted in a noticeable increase in the absorption spectrum of SnF_2, indicating the oxidation of SnF_2 by H_2O_2. **b** Representative UV-visible absorption spectra of Fer in combination with SnF_2 in the presence of H_2O_2 at different time points, as indicated, in 0.1 M sodium acetate buffer (pH 4.5). The absorption spectra of Fer+SnF_2, upon incubation with H_2O_2 at various time points, do not change appreciably when compared to Fer alone, even after 60 min of catalysis, suggesting Fer may prevent oxidation of SnF_2. Source data are available as a Source Data file.

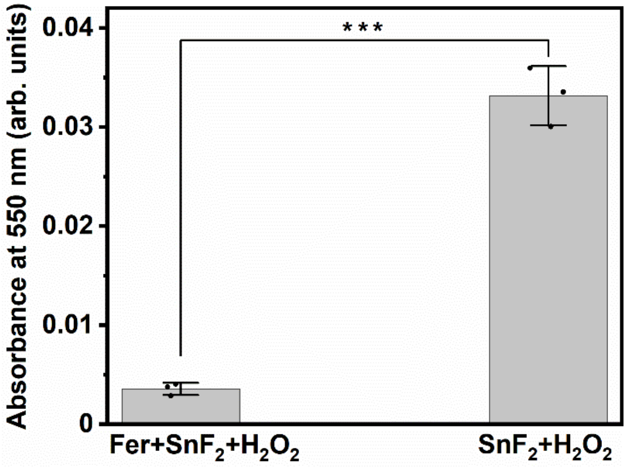

Supplementary Fig. 11. Stability study of SnF_2 with or without Fer after H_2O_2 treatment. Comparison of the absorbance of SnF_2 (0.1 mg/ml) at 550 nm in the presence of H_2O_2 (0.1%, v/v) with or without Fer (0.1 mg of Fe/ml) in 0.1 M sodium acetate buffer (pH 4.5). The absorbance of Fer was used as the background for the Fer+SnF_2+H_2O_2 group. The absorbance measurements were taken after 1 h incubation in the presence of H_2O_2. The data are presented as mean ± standard deviation (n=3 independent experiments). ***$p < 0.001$; one-way ANOVA followed by Tukey test. Source data are available as a Source Data file.

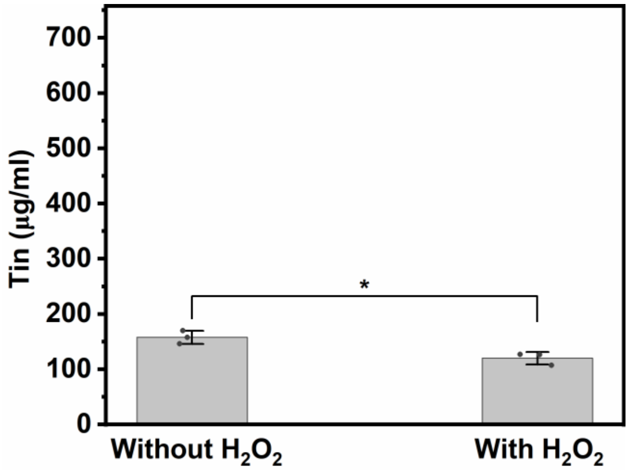

Supplementary Fig. 12. Assessment of free tin ions before and after H_2O_2 treatment. Comparison of the concentration of free tin ions in the filtrate when SnF_2 (1 mg/ml) was mixed with Fer (1 mg of Fe/ml) in 0.1 M sodium acetate buffer (pH 4.5) after 10 min incubation in the absence and presence of H_2O_2 (1%, v/v), as determined by ICP-OES. The data are presented as mean ± standard deviation (n=3 independent experiments). *$p < 0.05$; one-way ANOVA followed by Tukey test. Source data are available as a Source Data file.

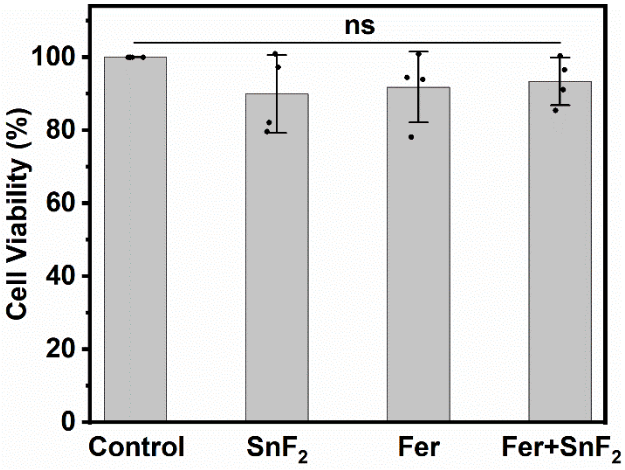

Supplementary Fig. 13. *In vitro* biocompatibility study via MTS assay. Effect of the combined treatment of Fer (1 mg of Fe/ml) and SnF_2 (250 ppm of F) on the cell viability of HGK cells. The data are presented as mean ± standard deviation (n=4 independent experiments). ns, nonsignificant; one-way ANOVA followed by Tukey test. Source data are available as a Source Data file.

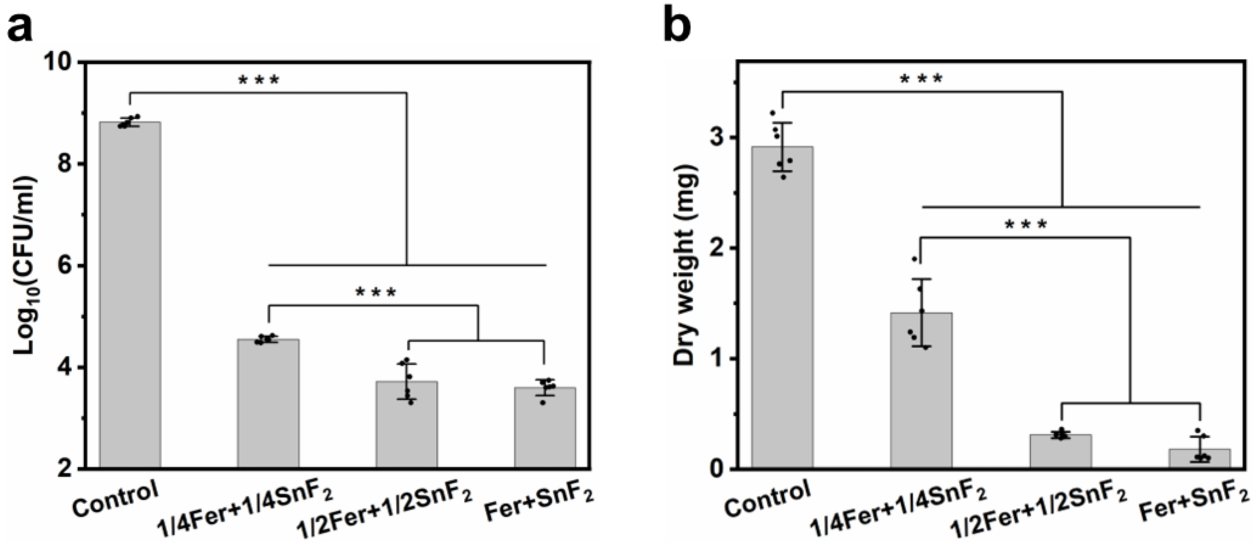

Supplementary Fig. 14. Antibiofilm study of the combined treatment of Fer and SnF_2 at different concentrations. The bacterial viability (**a**) and biofilm mass (**b**) with the varied concentration of Fer (0-1 mg of Fe/ml) and SnF_2 (0-250 ppm of F). 1/4Fer, 1/2Fer, and Fer indicate 0.25 mg of Fe/ml, 0.5 mg of Fe/ml, and 1 mg of Fe/ml, respectively. Similarly,1/4SnF_2, 1/2SnF_2, and SnF_2 indicate 62.5 ppm of F, 125 ppm of F, and 250 ppm of F, respectively. The data are presented as mean ± standard deviation (n=6; 3 independent experiments with two replicates). ***$p < 0.001$; one-way ANOVA followed by Tukey test. Source data are available as a Source Data file.

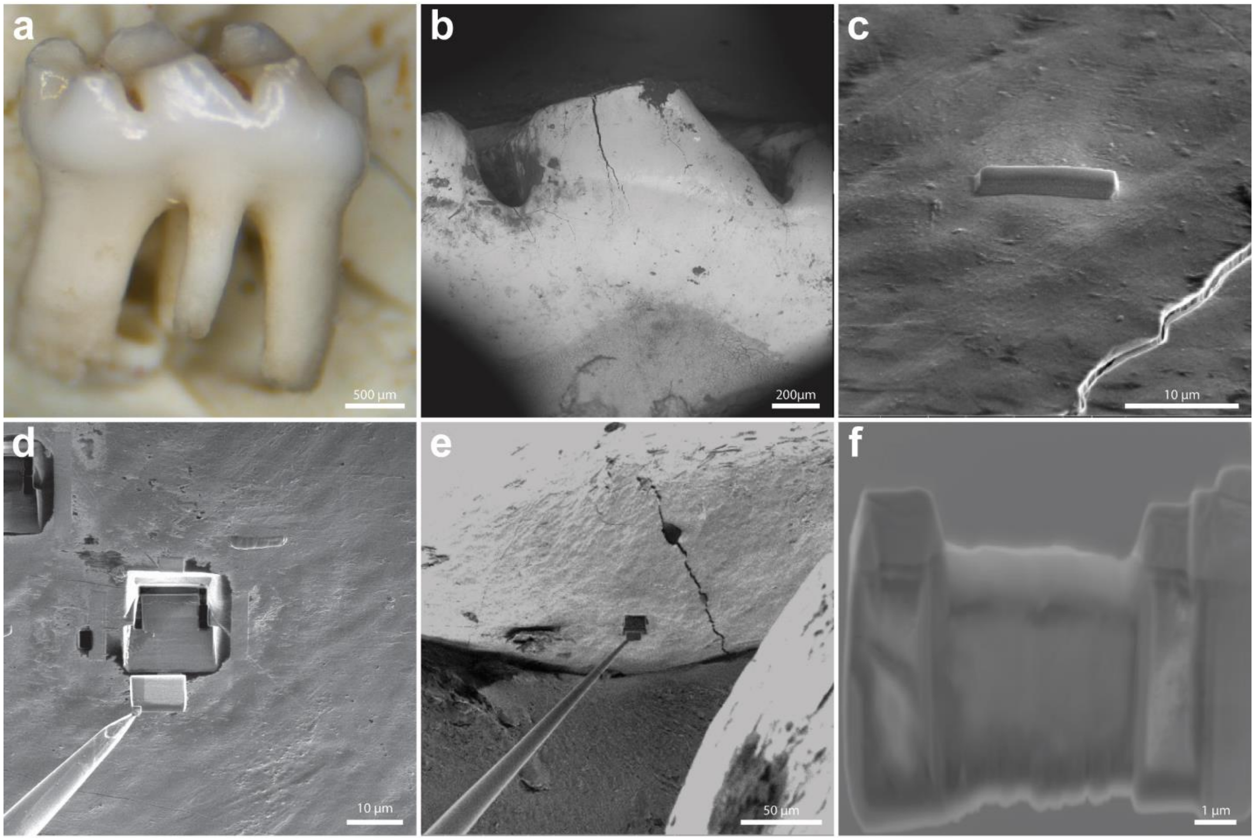

Supplementary Fig. 15. Workflow for lift-out and thinning of lamellae from M1 rat molars for analysis by STEM-EDS and STEM-EELS. a Buccal aspect of whole M1 molar. **b** SEM image of the middle cusp of molar in (**a**). **c** SEM image of enamel surface after deposition of a protective strap of FIB-Pt. **d**, **e** SEM images of enamel surface with lamella attached to micro-manipulator after lift-out. **f** SEM image of lamella after thinning.

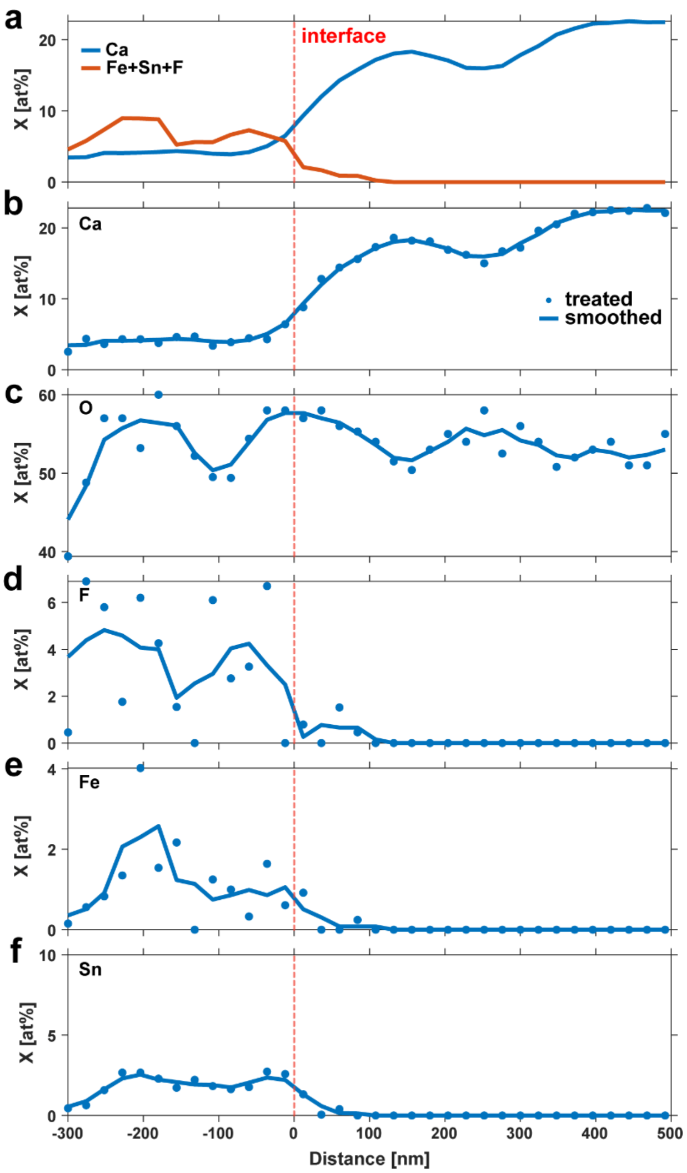

Supplementary Fig. 16. Elemental composition of the surface of a rat M1 molar treated with Fer+SnF_2 as assessed by STEM-EELS. a-f Plot of the mean mole fraction of Ca (blue line), and of the sum of the mean mole fractions of Fe, Sn, and F (red line, **a**), and plot of mean mole fractions of Ca (**b**), O (**c**), F (**d**), Fe (**e**), and Sn (**f**) vs. distance in the direction normal to the EES. The distance axis is referenced to the approximate position of the interface between the Fe/Sn/F-rich layer and the underlying enamel. For **b-f**, solid circles indicate the mean mole fraction at a given distance, and lines indicate the moving average of the mole fraction with span 3 (denoted as "smoothed" in legend). Note that the data shown here was generated from a separately prepared sample extracted from the same tooth shown in Fig. 5f. Source data are available as a Source Data file.

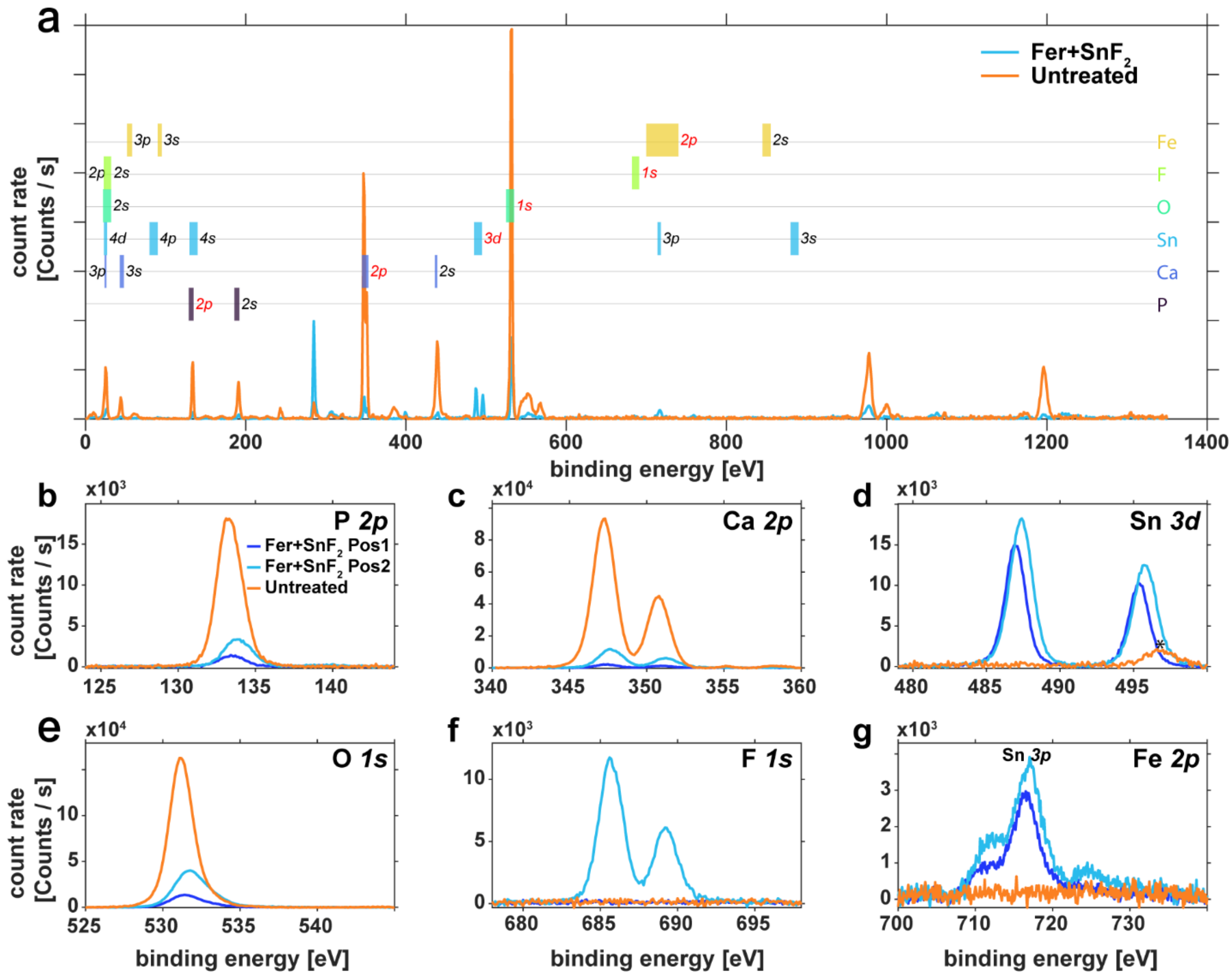

Supplementary Fig. 17. Example XPS spectra of mature rat molar enamel. a Survey spectra of teeth from Fer+SnF_2-treated (blue, after a sputtering time of t_s = 1000 s) and untreated (orange, t_s = 0 s) animals. Colored bands indicate binding energy ranges for selected elements. **b-g** Detailed, background-corrected spectra for the P *2p* (**b**), Ca *2p* (**c**), Sn *3d* (**d**), O *1s* (**e**), F *1s* (**f**), and Fe *2p* (**g**) spectral lines recorded on opposite sides of a maxillary molar of a Fer+SnF_2-treated animal (Pos 1, dark blue; Pos 2, light blue; t_s = 1000 s), and at the surface of a tooth from an untreated animal (untreated, orange). Note that the feature at ~496 keV in the untreated sample in (**d**) likely corresponds to the NaKL1 line (asterisk) and that the Sn *3p* and Fe *2p* lines overlap (**g**). For quantification, the contribution of the Fe *2p* line was isolated by fitting both lines. Source data are available as a Source Data file.

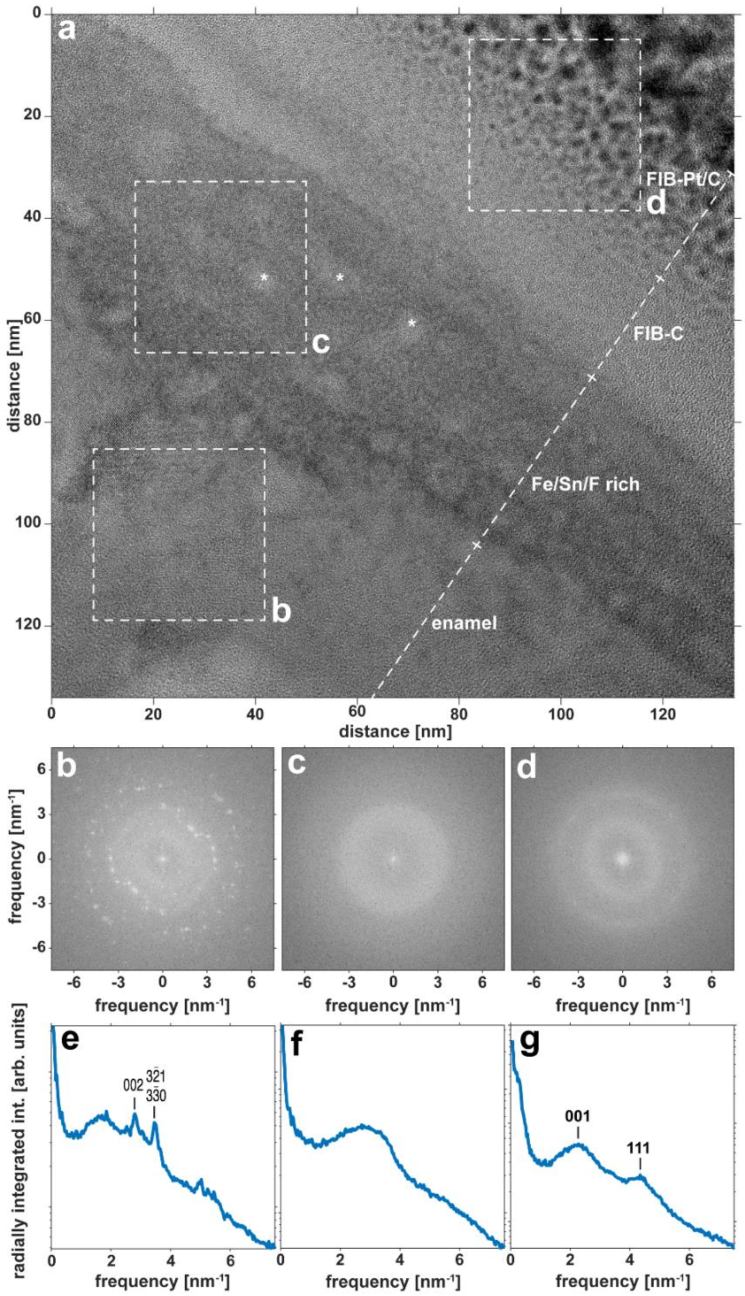

Supplementary Fig. 18. HRTEM analysis of coating on treated rat molars. a HRTEM image. Note that likely some beam damage is apparent in the Fe/Sn/F-rich layer (asterisks). **b-d** Magnitude of 2D Fast Fourier Transforms (FFTs) of areas indicated in (**a**). **e-g** Plots of radial integrals of FFTs (**e**) in (**b**), (**f**) in (**c**), and (**g**) in (**d**). Spatial frequencies consistent with d-spacing for the $\{002\}$, $\{3\bar{2}1\}$, and $\{3\bar{3}0\}$ sets of planes of hydroxylapatite are indicated in (**e**), and those consistent with d-spacing for the $\{001\}$ and $\{111\}$ sets of planes in Pt are indicated in (**g**). Source data are available as a Source Data file.

Supplementary Table 1. Hydrodynamic diameter (in nm) of Fer with or without SnF_2 in DI water at various incubation times. The data are presented as mean ± standard deviation. The mean and standard deviation were calculated from the three means.

Incubation Time (h)	Fer	Fer+SnF_2
0	35.0 ± 0.16	26.0 ± 5.3
1	34.4 ± 1.3	28.7 ± 1.4
2	32.9 ± 0.42	41.0 ± 2.7
4	35.0 ± 3.3	33.9 ± 2.0
6	34.1 ± 1.7	38.3 ± 1.3
24	31.9 ± 0.43	34.1 ± 0.11

Supplementary Table 2. Zeta potential (in mV) of Fer with or without SnF_2 in DI water at various incubation times. The data are presented as mean ± standard deviation. The mean and standard deviation were calculated from three means.

Incubation Time (h)	Fer	Fer+SnF_2
0	-48.5 ± 1.4	-10.5 ± 0.84
1	-42.0 ± 4.1	-11.0 ± 1.1
2	-42.8 ± 7.3	-10.6 ± 0.92
4	-45.7 ± 2.1	-10.6 ± 0.73
6	-41.1 ± 5.0	-10.1 ± 0.61
24	-39.4 ± 2.5	-11.8 ± 1.2
