# Supplementary material for: Iron oxide nanozymes stabilize stannous fluoride for targeted biofilm killing and synergistic oral disease prevention
Source: Nat Commun. 2023 Sep 29;14:6087. doi: 10.1038/s41467-023-41687-8 (PMC10541875; doi:10.1038/s41467-023-41687-8)
Supplement: Supplementary file 2 — Reporting Summary [file 41467_2023_41687_MOESM2_ESM.pdf]

nature portfolio

Corresponding author(s): David P. Cormode & Hyun Koo

Last updated by author(s): 2023.08.24

Reporting Summary

Nature Portfolio wishes to improve the reproducibility of the work that we publish. This form provides structure for consistency and transparency in reporting. For further information on Nature Portfolio policies, see our Editorial Policies and the Editorial Policy Checklist.

Statistics

For all statistical analyses, confirm that the following items are present in the figure legend, table legend, main text, or Methods section.

n/a	Confirmed	
☐	☒	The exact sample size (*n*) for each experimental group/condition, given as a discrete number and unit of measurement
☐	☒	A statement on whether measurements were taken from distinct samples or whether the same sample was measured repeatedly
☐	☒	The statistical test(s) used AND whether they are one- or two-sided<br>*Only common tests should be described solely by name; describe more complex techniques in the Methods section.*
☒	☐	A description of all covariates tested
☒	☐	A description of any assumptions or corrections, such as tests of normality and adjustment for multiple comparisons
☐	☒	A full description of the statistical parameters including central tendency (e.g. means) or other basic estimates (e.g. regression coefficient) AND variation (e.g. standard deviation) or associated estimates of uncertainty (e.g. confidence intervals)
☐	☒	For null hypothesis testing, the test statistic (e.g. *F*, *t*, *r*) with confidence intervals, effect sizes, degrees of freedom and *P* value noted<br>*Give P values as exact values whenever suitable.*
☒	☐	For Bayesian analysis, information on the choice of priors and Markov chain Monte Carlo settings
☒	☐	For hierarchical and complex designs, identification of the appropriate level for tests and full reporting of outcomes
☒	☐	Estimates of effect sizes (e.g. Cohen's *d*, Pearson's *r*), indicating how they were calculated

Our web collection on statistics for biologists contains articles on many of the points above.

Software and code

Policy information about availability of computer code

Data collection	No software has been used to collect data because the data presented in this study were collected experimentally.
Data analysis	We have discussed them in the manuscript (if applicable), which include ImageJ (version 1.48), Excel 2016, Origin 2022, Origin 2023, Avantage, Matlab r2022b, QIIME2 v19.4, and R environment (version 4.0.3).

For manuscripts utilizing custom algorithms or software that are central to the research but not yet described in published literature, software must be made available to editors and reviewers. We strongly encourage code deposition in a community repository (e.g. GitHub). See the Nature Portfolio guidelines for submitting code & software for further information.

Data

Policy information about availability of data

All manuscripts must include a data availability statement. This statement should provide the following information, where applicable:

- Accession codes, unique identifiers, or web links for publicly available datasets
- A description of any restrictions on data availability
- For clinical datasets or third party data, please ensure that the statement adheres to our policy

16S rRNA sequencing data is available in the public repository NCBI under the accession number PRJNA914620 [https://www.ncbi.nlm.nih.gov/bioproject/914620]. All the other data that support the findings of this study are available in the main text or the supplementary information. Source data are provided with this paper.

Human research participants

Policy information about studies involving human research participants and Sex and Gender in Research.

Reporting on sex and gender	No human research participant
Population characteristics	No human research participant
Recruitment	No human research participant
Ethics oversight	No human research participant

Note that full information on the approval of the study protocol must also be provided in the manuscript.

Field-specific reporting

Please select the one below that is the best fit for your research. If you are not sure, read the appropriate sections before making your selection.

[x] Life sciences [ ] Behavioural & social sciences [ ] Ecological, evolutionary & environmental sciences

For a reference copy of the document with all sections, see nature.com/documents/nr-reporting-summary-flat.pdf

Life sciences study design

All studies must disclose on these points even when the disclosure is negative.

Sample size	Sample sizes were chosen according to the standard in the field. For animal study, 16 rats were used for each treatment group.
Data exclusions	For animal study, 2-3 outliers from each group were removed for analyses.
Replication	For animal study, 16 rats were used for each treatment group to ensure the reproducibility of our work. All other experiments were preformed at least 3 times unless otherwise stated. All attempts at replication were successful.
Randomization	For animal study, the animals were orally infected with S. mutans UA159, and their infections were confirmed at 21 days via oral swabbing. All infected pups were randomly placed into five treatment groups, and their teeth were treated twice daily.
Blinding	Determination of the caries score of the jaws was performed by a calibrated examiner who was blinded for the study by using codified samples.

Reporting for specific materials, systems and methods

We require information from authors about some types of materials, experimental systems and methods used in many studies. Here, indicate whether each material, system or method listed is relevant to your study. If you are not sure if a list item applies to your research, read the appropriate section before selecting a response.

Materials & experimental systems

n/a	Involved in the study	
x		Antibodies
	x	Eukaryotic cell lines
x		Palaeontology and archaeology
	x	Animals and other organisms
x		Clinical data
x		Dual use research of concern

Methods

n/a	Involved in the study	
x		ChIP-seq
x		Flow cytometry
x		MRI-based neuroimaging

Eukaryotic cell lines

Policy information about cell lines and Sex and Gender in Research

Cell line source(s)	HGK cells were kindly provided by the laboratory of Dana T. Graves (School of Dental Medicine, University of Pennsylvania).
Authentication	Cell line was authenticated by inspecting morphology and growth of cells.
Mycoplasma contamination	Cells were not tested for mycoplasma contamination.
Commonly misidentified lines (See ICLAC register)	Commonly misidentified lines were not used in this study.

Animals and other research organisms

Policy information about studies involving animals; ARRIVE guidelines recommended for reporting animal research, and Sex and Gender in Research

Laboratory animals	15 days-old specific pathogen free Sprague-Dawley rat pups were purchased with their dams from Harlan Laboratories (Madison, WI, USA).
Wild animals	The study did not involve any wild animals.
Reporting on sex	No sex or gender based analyses were performed in this study.
Field-collected samples	The study did not involve samples collected from the field.
Ethics oversight	This research was reviewed and approved by the University of Pennsylvania Institutional Animal Care and Use Committee (IACUC #805529).

Note that full information on the approval of the study protocol must also be provided in the manuscript.
